# Effects of supplemental fulvic acid on survival, growth performance, digestive ability and immunity of large yellow croaker (*Larimichthys crocea*) larvae

**DOI:** 10.3389/fphys.2023.1159320

**Published:** 2023-03-23

**Authors:** Chenxiang Zhang, Yongtao Liu, Chuanwei Yao, Jianmin Zhang, Yuntao Wang, Jiahui Liu, Yucong Hong, Kangsen Mai, Qinghui Ai

**Affiliations:** ^1^ Key Laboratory of Aquaculture Nutrition and Feed (Ministry of Agriculture and Rural Affairs), Key Laboratory of Mariculture (Ministry of Education), Ocean University of China, Qingdao, China; ^2^ Laboratory for Marine Fisheries Science and Food Production Processes, Qingdao National Laboratory for Marine Science and Technology, Qingdao, China

**Keywords:** fulvic acid, large yellow croaker, larval nutrition, intestinal morphology, immune response

## Abstract

A 30-day feeding trial was designed to evaluate the effect of supplemental fulvic acid (FA) on survival, growth performance, digestive ability and immunity of large yellow croaker (*Larimichthys crocea*) larvae (initial body weight 11.33 ± 0.57 mg). Four isonitrogenous and isolipids diets containing 0.00%, 0.01%, 0.02% and 0.04% FA were formulated, respectively. Results showed that the supplementation of 0.04% FA significantly improved survival rate of large yellow croaker larvae. Meanwhile, supplemental FA significantly increased final body weight and specific growth rate. Based on the specific growth rate, the optimal supplementation was 0.0135% FA. Larvae fed the diet with 0.01% FA had significantly higher villus height than the control. The supplementation of 0.01%–0.02% FA significantly increased the muscular thickness of intestine. Moreover, supplementation of FA significantly increased mRNA expression of intestinal epithelial proliferation and barrier genes (*pcna*, *zo-1* and *zo-2*). Diets supplemented with 0.02%–0.04% FA significantly increased the activity of trypsin in the intestinal segment, while 0.01%–0.02% FA significantly increased the activity of trypsin in the pancreatic segment. Compared with the control, supplementation of FA remarkably increased activities of alkaline phosphatase and leucine aminopeptidase in the brush border membrane of intestine. Larvae fed the diet with 0.01% FA significantly increased activities of lysozyme and total nitric oxide synthase. Furthermore, the supplementation of 0.01% to 0.02% FA significantly decreased the mRNA expression of pro-inflammatory cytokines (*tnf-α* and *il-6*). Concurrently, supplemental FA significantly increased anti-inflammatory cytokine (*il-10*) mRNA expression level. In conclusion, this study indicated that the supplementation of FA could improve the survival rate and growth performance of larvae by promoting intestinal development, digestive enzymes activities and innate immunity.

## 1 Introduction

Production of larvae is a critical bottleneck restricting the development of marine fish farming (Zhang et al., 2023). Inadequate digestive system and low immunity are the chief causes of high mortality of larvae (Comabella et al., 2013; Rojo-Cebreros et al., 2018). Therefore, finding ecofriendly feed additives to promote the maturation of digestive function and the development of immune system in larval stage is a powerful way for aquacultural flourish.

Fulvic acid (FA) is an organic substance found in humus with the lowest molecular size (around 2000 Da) but the highest activity (Isam et al., 2005; Plaza et al., 2005). Due to the various functional groups of FA, it has beneficial functions such as anti-inflammatory, antibacterial, stimulating the absorption of microelements, and enhancing immunostimulatory properties (Van Rensburg, 2015; Hullár et al., 2018; Goenadi, 2021). Studies in mammals have shown that FA has potential to prevent or treat diseases, such as diabetes and neurodegenerative disorders (Winkler and Ghosh, 2018; Dominguez-Meijide et al., 2020). In livestock and poultry, FA has been shown to improve lipid metabolism, intestinal morphology and meat quality (Bai et al., 2013; Chang et al., 2014; Taklimi et al., 2012). Researches of FA in aquatic animals have made progress in recent years. Studies proved that dietary FA can promote growth and digestive ability in *Carassius auratus* (Wu et al., 2016), regulate intestinal microbiota of *Paramisgurnus dabryanus* (Gao et al., 2017), improve antioxidant capacity of *Oreochromis niloticus* (Neamatallah et al., 2022), and enhance non-specific immunity of *Procambarus clarkia* (Zhang, 2018). However, the effects of supplemental FA on intestinal development and immune function of marine fish larvae have not been reported.

Large yellow croaker (*Larimichthys crocea*) is one of the main economic fish in China (He et al., 2022). Lots of studies in nutrition requirements, digestive physiology and feed manufacturing technology of larvae have been reported (Ai et al., 2008; Zhao et al., 2013; Cai et al., 2017; Liu et al., 2022). However, there is no study on effects of FA on digestive system maturation and immunity of large yellow croaker larvae. Therefore, this experiment was conducted to investigate the effects of supplemental FA on survival, growth performance, digestive capacity and immunity of large yellow croaker larvae.

## 2 Materials and methods

### 2.1 Diets formulation

Four isonitrogenous (crude protein 50.4%) and isolipidic (crude lipid 17.2%) experimental diets supplemented with 0.00%, 0.01%, 0.02% and 0.04% FA (purity 85%, Shanghai Macklin Biochemical Co., Ltd), respectively (Table 1). Each experimental feed ingredient was crushed to 150 mesh, then FA was mixed with powdered ingredients in the order of addition from less to more. The remaining ingredients were added to an electric blender and stirred for 30 min until a uniform, viscous pellet was formed. The pellet was made into 1 mm micro-diets, and then oven-baked for about 8 h at 50°C. The dried feed was broken and filtrated to obtain two micro-diets with particle sizes of 280–450 μm and 450–600 μm. The dried feed was stored at −20°C for use in subsequent experiments. Diets of 280–450 μm were fed to larvae 17–32 days after hatching (DAH), and diets of 450–600 μm were fed afterwards.

**TABLE 1:** Formulation and proximate composition of the experimental diets (% dry weight).

Ingredient% dry diet	Diets (FA%)			
	Control (0.00)	Diet 1 (0.01)	Diet 2 (0.02)	Diet 3 (0.04)
White fish meal^1^	36.86	36.86	36.86	36.86
Krill meal^1^	21.02	21.02	21.02	21.02
Squid meal^1^	14.27	14.27	14.27	14.27
Yeast extract^1^	2.00	2.00	2.00	2.00
Microcrystalline cellulose	0.04	0.03	0.02	0
Strong flour	4.00	4.00	4.00	4.00
α-starch	4.00	4.00	4.00	4.00
Sodium alginate	2.00	2.00	2.00	2.00
Vitamin premix^2^	1.50	1.50	1.50	1.50
Mineral premix^2^	1.00	1.00	1.00	1.00
Ascorbyl polyphosphate	0.20	0.20	0.20	0.20
Mold inhibitor	0.05	0.05	0.05	0.05
Antioxidant	0.05	0.05	0.05	0.05
Choline chloride	0.20	0.20	0.20	0.20
Fish oil	8.00	8.00	8.00	8.00
Soybean lecithin^3^	5.00	5.00	5.00	5.00
Fulvic acid^4^	0	0.01	0.02	0.04
Analyzed nutrients composition (dry matter basis)				
Crude protein (%)	50.05	51.18	49.97	50.35
Crude lipid (%)	16.84	17.60	17.55	16.91
Moisture (%)	6.21	6.38	6.65	6.56

^1^ Ingredients for feed were purchased from Great Seven Biotechnology Co., ltd in shandong, China; The feed compositions were referred to Zhang et al. (2023).

^2^ The composition of mineral and vitamin premix (g/kg) was referred to Zhang et al. (2023).

^3^ Commercially available from Beijing Huaxia Houde Co., Ltd. (Beijing, China).

^4^ Fulvic acid was purchased from Shanghai Macklin Biochemical Co., Ltd. (Shanghai, China). The purity was ≥85.0%.

### 2.2 Experimental procedures

The experiment was carried out at the Ningbo Marine and Fishery Science and Technology Innovation Base in Zhejiang Province. The experimental objects were artificially hatched large yellow croaker larvae from the same batch. Larvae were fed with rotifers (*Brachionus plicatilis*) from three to eight DAH. From 6 to 11 DAH, the larvae were fed with brine shrimp (*Artemia nauplii*). From 10 to 17 DAH, larvae were feed copepod (*Calanus sinicus*) and micro-diet, with the proportion of diet gradually increasing until 17 DAH, when the experimental diet was fed exclusively. In the experiment, larvae (mean body weight 11.33 ± 0.57 mg) were randomly assigned to 12 cylindrical white barrels (water volume 220 L), with a density of 3,000 individuals per barrel. Larvae were fed the experimental diet, and four treatment groups were set up with three replicates per treatment. During the rearing period (17–47 DAH), the experimental feed was fed to larvae seven times a day (06:00, 09:00, 12:00, 15:00, 18:00, 21:00, 24:00) until satiation. The rearing conditions are strictly controlled and water temperature, pH, and salinity were ranging from 23°C to 26°C, 7.8 to 8.2, 21‰–25‰, respectively. Approximately 150%–200% of the water was replaced daily.

### 2.3 Sample collection

At the beginning, fifty larvae of 17 DAH were randomly selected from each holding bucket, and initial body weight (IBW) and initial body length (IBL) were measured using an analytical balance and vernier caliper, respectively. At the end of the feeding trial, larvae were fasted for 24 h to empty their digestive tracts. Larvae were counted and survival rates (SR) were calculated for each barrel. Fifty larvae from each tank were randomly collected to measure final body weight (FBW) and final body length (FBL). Fifty larvae were randomly selected from each barrel and dissected under a dissecting microscope at 0°C to get the pancreatic and intestinal segments (PS and IS) for the detection of digestive enzyme activity (Cahu and Infante, 1994). Forty larvae were randomly selected from each barrel and dissected at 0°C to get visceral masses (VM) for analysis immune enzyme activities. Ninety larvae were randomly selected from each barrel and dissected under a dissecting microscope at 0°C in a sterile environment. The whole intestine and liver were separated and stored in RNase-free centrifuge tubes and immediately frozen in liquid nitrogen for real-time quantitative PCR analysis. Fifteen larvae from each treatment group were randomly selected and fixed in 4% paraformaldehyde for 24 h, then transferred to 75% alcohol for HE staining. After sampling, the remaining larvae were divided into 10 mL RNase-free centrifuge tubes and snap-frozen in liquid nitrogen for analysis of body composition.

### 2.4 Analytical methods

#### 2.4.1 Proximate composition analysis

Diets and larvae were oven baked at 105°C for 72 h to constant weight to determine water content. Crude protein content of diets and fish was determined by the Kjeltec nitrogen method (FOSS Kjeltec 8,400 Analyzer Uni, Sweden) and estimated by multiplying nitrogen by 6.25. Crude lipid content of diets and fish was examined by the Soxhlet extraction method (Soxhlet Extraction System B-801, Buchi 36,680, Switzerland).

#### 2.4.2 Intestinal morphology analysis

Intestinal sections were prepared according to the methods described in the published paper (Mai et al., 2005). In brief, larvae intestinal segments were dehydrated in a gradient, embedded in paraffin, sectioned, and immediately stained with hematoxylin and eosin solutions. Sections were observed under a light microscope (Leica DM3000 LED, Germany), and professional image analysis software (Image Pro Plus 6.0, United States) was used to measure the villus height, enterocyte cell height and muscular thickness.

#### 2.4.3 Digestive enzyme activities assay

The PS and IS samples were mixed and homogenized at a 1:9 ratio, centrifuged at 3,500 *g* for 15 min, and the supernatant was used for index determination. Purified brush border membranes (BBM) were obtained from homogenates of IS according to the method described by Crane et al. (1979). LAP activity was determined according to the method of Maroux et al. (1973). Trypsin, *α*-amylase, lipase and AKP activities were measured using commercial kits (Nanjing Jiancheng Bio-Engineering Institute, China). Total protein quantification (TP) was determined using the Beyotime BCA kit (P0011). All experimental procedures were performed in strict accordance with the instructions.

#### 2.4.4 Immune enzymes activities assay

The VM sample were mixed and homogenized at a 1:9 ratio, centrifuged at 3,500 *g* for 15 min, and the supernatant was used for index determination. LZM, TNOS and iNOS activities were measured using commercial kits (Nanjing Jiancheng Bio-Engineering Institute, China).

#### 2.4.5 RNA extraction and real-time quantitative PCR

Total RNA was extracted from the intestine or liver of larvae using RNAiso Plus (Takara Biotech, Japan). cDNA was reverse transcribed from the RNA using the Prime Script-RT kit (Takara, Japan). The primer sequences were designed and synthesized based on the corresponding sequences in published papers (Table 2). Real-time quantitative PCR was performed by referring to Zhang et al. (2023).

**TABLE 2:** Primers used for quantitative PCR.

Gene	Forward (5′–3′)	Reverse (5′–3′)	References
*occludin*	AGG​CTA​CGG​CAA​CAG​TTA​TG	GTG​GGT​CCA​CAA​AGC​AGT​AA	Zhang et al. (2023)
*zo-1*	TGT​CAA​GTC​CCG​CAA​AAA​TG	CAA​CTT​GCC​CTT​TGA​CCT​CT	Zhang et al. (2023)
*zo-2*	ACC​CGA​CCT​GTT​TGT​TAT​TG	ATGCCGTGCTTGCTGTC	Liu et al. (2020)
*pcna*	AGTTTGCCCGTATCTGCC	CTC​TTT​GTC​TAC​ATT​GCT​GGT​CT	Liu et al. (2020)
*odc*	GAG​CCA​GGT​CGC​TTC​TAT​G	CCGTGGTCCCTTCGTCT	Liu et al. (2020)
*ifn-γ*	TCA​GAC​CTC​CGC​ACC​ATC​A	GCA​ACC​ATT​GTA​ACG​CCA​CTT​A	Li et al. (2020)
*tnf-α*	ACA​CCT​CTC​AGC​CAC​AGG​AT	CCG​TGT​CCC​ACT​CCA​TAG​TT	Wang et al. (2022)
*il-1β*	CAT​AGG​GAT​GGG​GAC​AAC​GA	AGG​GGA​CGG​ACA​CAA​GGG​TA	Wang et al. (2022)
*il-6*	CGA​CAC​ACC​CAC​TAT​TTA​CAA​C	TCC​CAT​TTT​CTG​AAC​TGC​CTC​T	Li et al. (2020)
*il-10*	AGT​CGG​TTA​CTT​TCT​GTG​GTG	TGT​ATG​ACG​CAA​TAT​GGT​CTG	Li et al. (2020)
*β-actin*	GAC​CTG​ACA​GAC​TAC​CTC​ATG	AGT​TGA​AGG​TGG​TCT​CGT​GGA	Liu et al. (2020)

Abbreviation: *zo-1*, zonula occludens-1; *zo-2*, zonula occludens-2; *pcna*, proliferating cell nuclear antigen; *odc*, ornithine decarboxylase; *ifn-γ*, interferon γ; *tnf-α*, tumor necrosis factor α; *il-1β*, interleukin-1β; *il-6*, interleukin-6; *il-10*, interleukin-10.

### 2.5 Calculations and statistical analysis

The following calculations were performed:

$$Survival\;rate\;\left(SR,\%\right)=N_{t}\times 100/N_{0}$$

$$Specific\;growth\;rate\;\left(SGR,\%{day}^{-1}\right)=\left({LnW}_{t}\;–\;{LnW}_{0}\right)\times 100/d$$

Where N_t_ and N_0_ were the final and initial number of larvae, respectively; W_t_ and W_0_ were the final and initial body weights, respectively; d was the experimental period in days.

Statistical analysis was performed in SPSS 26.0 (SPSS Inc., United States). The data were analyzed by the one-way analysis of variance (ANOVA) followed by Tukey’s multiple-range test. Statistically significant differences were determined using *p* < 0.05. Results were presented as mean ± S.E.M. (Standard error of means).

## 3 Results

### 3.1 Survival, growth performance and body composition

**FIGURE 1:**
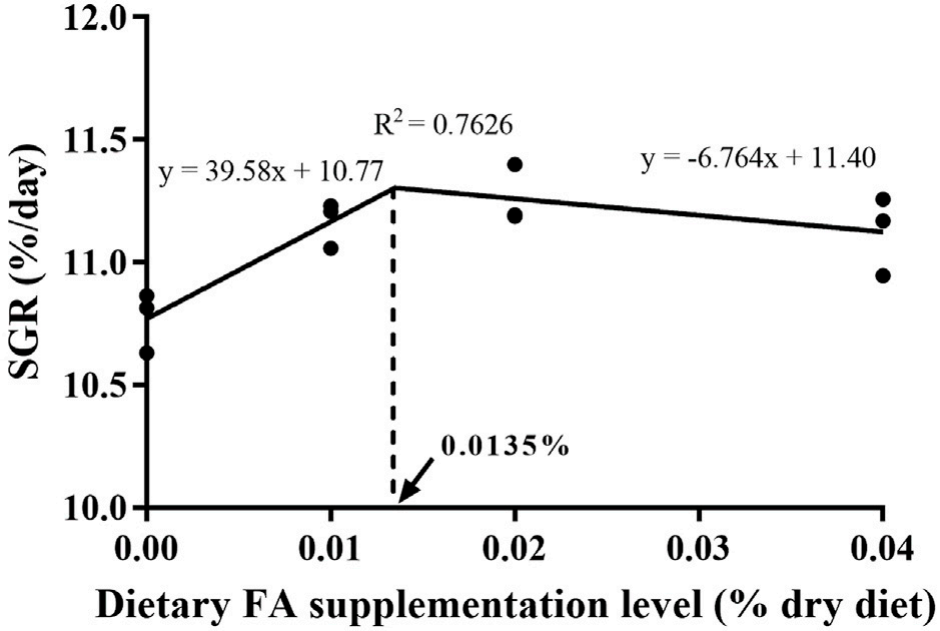
Relationship of specific growth rate (SGR, %/d) with supplemental FA levels of large yellow croaker larvae.

After 30 days, the supplementation of FA significantly improved the survival rate and growth performance of larvae (Table 3) (*p* < 0.05). When the FA supplementation reached 0.04%, the survival rate significantly increased from 20.64% to 23.48% (*p* < 0.05). Supplementation of 0.01%–0.04% FA increased FBL, FBW and SGR of larvae significantly (*p* < 0.05). The broken-line analysis for SGR indicated that the maximum growth of larvae appeared in the supplementation of 0.0135% FA (Figure 1). There were no significant differences in moisture, crude lipid and crude protein contents among supplementary treatments (*p* > 0.05) (Table 4).

**TABLE 3:** Effects of supplemental fulvic acid on survival rate and growth performance of large yellow croaker larvae (Means ± S.E.M., n = 3)^1^

Parameters	Diets (FA%)			
	Control (0.00)	Diet 1 (0.01)	Diet 2 (0.02)	Diet 3 (0.04)
Initial body length (mm)	8.77 ± 0.22	8.77 ± 0.22	8.77 ± 0.22	8.77 ± 0.22
Final body length (mm)	24.03 ± 0.14^b^	25.69 ± 0.12^a^	25.93 ± 0.41^a^	25.67 ± 0.30^a^
Initial body weight(mg)	11.33 ± 0.57	11.33 ± 0.57	11.33 ± 0.57	11.33 ± 0.57
Final body weight(mg)	286.89 ± 8.59^b^	323.00 ± 7.37^a^	332.33 ± 9.90^a^	319.22 ± 12.46^a^
Specific growth rate(%/day)	10.77 ± 0.10^b^	11.17 ± 0.08^a^	11.26 ± 0.10^a^	11.12 ± 0.13^a^
Survival rate(%)	20.64 ± 0.85^b^	20.36 ± 1.04^b^	20.72 ± 0.77^b^	23.48 ± 0.51^a^

^1^ Tukey’s test showed significant differences in the data without the same superscript letter in the same row (*p* < 0.05).

**TABLE 4:** Effects of supplemental fulvic acid on body composition of large yellow croaker larvae (Means ± S.E.M., n = 3)^1^.

Parameters	Diets (FA%)			
	Control (0.00)	Diet 1 (0.01)	Diet 2 (0.02)	Diet 3 (0.04)
Crude protein (%/w. w.^2^)	8.35 ± 1.21	8.58 ± 0.11	8.48 ± 0.60	8.40 ± 0.75
Crude lipid (%/w. w.)	3.77 ± 0.09	3.82 ± 0.12	3.76 ± 0.13	3.79 ± 0.16
Moisture (%)	84.12 ± 0.42	84.00 ± 0.21	84.27 ± 0.49	84.10 ± 0.61

^1^ Tukey’s test showed significant differences in the data without the same superscript letter in the same row (*p* < 0.05).

^2^ w.w., wet weight.

### 3.2 Intestinal morphology

**FIGURE 2:**
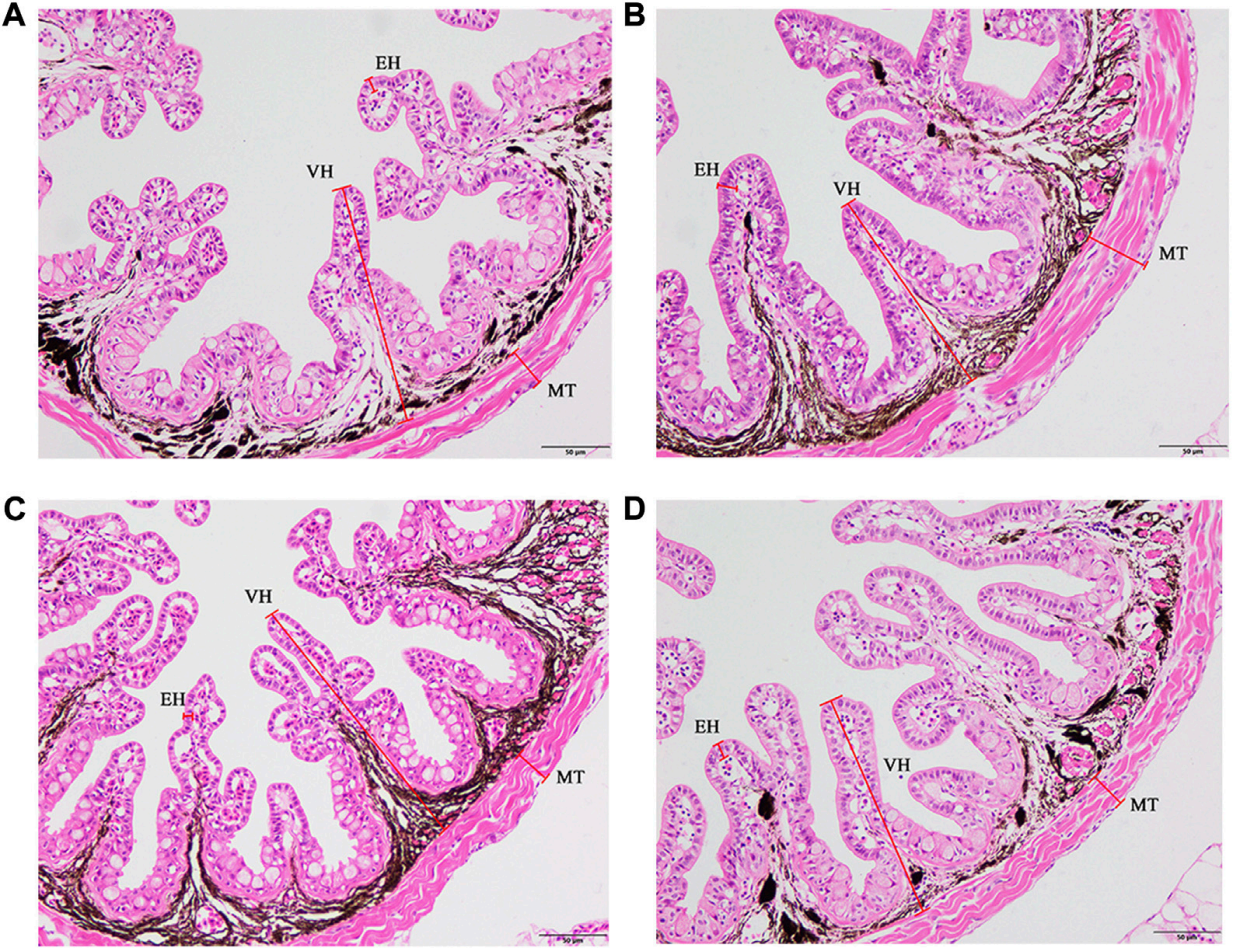
Effects of supplemental fulvic acid on intestinal morphology of large yellow croaker larvae. Control group (0.00%) **(A)**; 0.01% FA group **(B)**; 0.02% FA group **(C)**; 0.04% FA group **(D)**. MT, muscular thickness; VH, villus height; EH, enterocyte height (HE staining; Scale bar = 50 μm).

The supplementation of FA could improve the intestinal morphology of larvae (Figure 2). With an increase in FA supplementation, the VH, MT and EH increased firstly and then decreased. VH was significantly increased when FA supplementation was 0.01%. Compared with the control, the difference of MT was statistically significant when the supplemental level was 0.01%–0.02% (*p* < 0.05) (Table 5). However, no significant change of the EH was observed in the intestinal tract of larvae (*p* > 0.05) (Table 5).

**TABLE 5:** Effects of supplemental fulvic acid on micromorphology of the intestine of large yellow croaker larvae (Means ± S.E.M., n = 3)^1^.

Parameters	Diets (FA%)			
	Control (0.00)	Diet 1 (0.01)	Diet 2 (0.02)	Diet 3 (0.04)
Villus height (μm)	164.53 ± 23.14^b^	224.27 ± 29.92^a^	204.46 ± 21.85^ab^	197.01 ± 21.59^ab^
Enterocyte height (μm)	9.19 ± 1.15	10.59 ± 1.80	9.35 ± 2.21	9.65 ± 2.67
Muscular thickness (μm)	22.79 ± 3.36^b^	32.11 ± 4.60^a^	29.84 ± 3.61^a^	28.37 ± 2.48^ab^

^1^ Tukey’s test showed significant differences in the data without the same superscript letter in the same row (*p* < 0.05).

### 3.3 Expression of proliferation-related and barrier-related genes in intestinal cell

**FIGURE 3:**
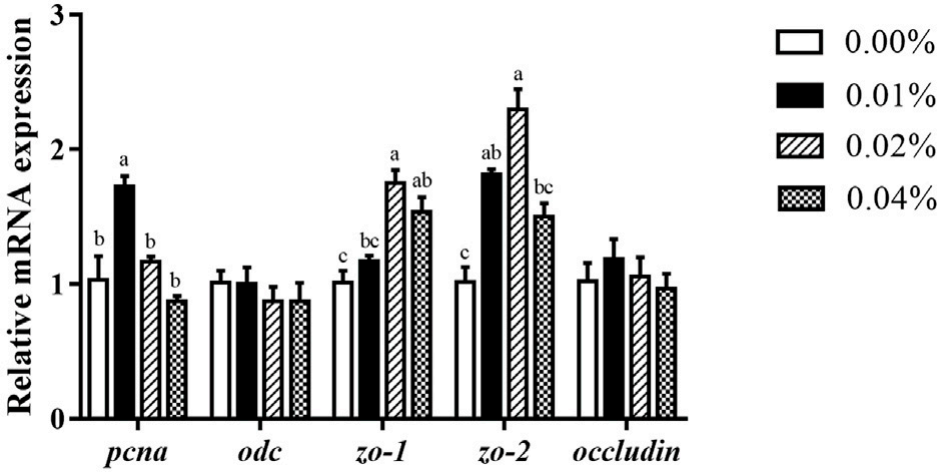
Effects of supplemental fulvic acid on intestinal cell proliferation-related and barrier-related genes mRNA expression in intestine of large yellow croaker larvae (mean ± S.E.M, n = 3). *Pcna*, proliferating cell nuclear antigen; *odc*, ornithine decarboxylase; *zo-1*, zonula occludens-1; *zo-2*, zonula occludens-2. Bars with different letters showed a significant difference (*p* < 0.05, Tukey’s test).

Compared to the control, supplementation of 0.02%–0.04% FA had notably higher mRNA expression of *zo-1* (*p* < 0.05) (Figure 3). The *zo-2* mRNA expression of larvae with 0.01%–0.02% FA supplementation was significantly higher than the control (*p* < 0.05) (Figure 3). Meanwhile, supplementation of 0.01% FA significantly increased the *pcna* mRNA expression (*p* < 0.05) (Figure 3). However, supplemental FA had no significant effect on the mRNA expression of *occludin* and *odc* (*p* > 0.05) (Figure 3).

### 3.4 Digestive enzyme activity

The supplementation of FA increased the activity of digestive enzyme (Table 6). Supplemental FA increased activities of trypsin in IS and PS significantly (*p* < 0.05). However, supplemental FA did not affect try-IS/(PS + IS) ratio of larvae significantly (*p* > 0.05). Meanwhile, compared with the control, activities of amylase and lipase were increased but the differences among treatments were not significant (*p* > 0.05).

**TABLE 6:** Effects of supplemental fulvic acid on activities of digestive enzymes of large yellow croaker larvae (Means ± S.E.M, n = 3)^1^.

Parameters		Diets (FA%)			
		Control (0.00)	Diet 1 (0.01)	Diet 2 (0.02)	Diet 3 (0.04)
Trypsin (U/mg protein)	IS^2^	254.91 ± 5.79^b^	272.96 ± 6.18^b^	343.90 ± 9.15^a^	315.76 ± 14.32^a^
	PS^2^	267.29 ± 20.02^c^	329.35 ± 5.99^ab^	382.65 ± 20.73^a^	322.71 ± 22.59^bc^
	Try-IS/(PS + IS)	0.49 ± 0.01	0.45 ± 0.01	0.47 ± 0.02	0.49 ± 0.02
Amylase (U/mg protein)	IS	0.050 ± 0.01	0.051 ± 0.01	0.063 ± 0.00	0.065 ± 0.00
	PS	0.034 ± 0.00	0.031 ± 0.00	0.038 ± 0.00	0.035 ± 0.00
Lipase (mU/mg protein)	IS	1.58 ± 0.09	1.84 ± 0.14	1.78 ± 0.14	1.50 ± 0.16
	PS	1.64 ± 0.18	1.87 ± 0.14	1.90 ± 0.16	1.68 ± 0.18

^1^ Tukey’s test showed significant differences in the data without the same superscript letter in the same row (*p* < 0.05).

^2^ PS, pancreatic segments; IS, intestinal segments.

The results suggested that activities of AKP and LAP were significantly higher when supplemented with 0.01%–0.04% FA than in the control (*p* < 0.05) (Table 7). With an increase in FA supplemental level, AKP and LAP activities initially increased and then decreased, with the highest activity observed when the supplemental amount was 0.01%.

**TABLE 7:** Effects of supplemental fulvic acid on activities of AKP and LAP in BBM^2^ of large yellow croaker larvae (Means ± S.E.M, n = 3)^1^.

Parameters	Diets (FA%)			
	Control (0.00)	Diet 1 (0.01)	Diet 2 (0.02)	Diet 3 (0.04)
AKP^2^ (mU/mg protein)	0.26 ± 0.04^c^	0.62 ± 0.05^a^	0.43 ± 0.03^b^	0.47 ± 0.02^b^
LAP^2^ (mU/mg protein)	9.16 ± 0.62^c^	21.52 ± 1.04^a^	16.06 ± 1.23^b^	15.08 ± 1.26^b^

^1^ Tukey’s test showed significant differences in the data without the same superscript letter in the same row (*p* < 0.05).

^2^ AKP, Alkaline-phosphatase; LAP, Leucine-aminopeptidase; BBM, brush border membrane.

### 3.5 Non-specific immune-related enzyme activity

**FIGURE 4:**
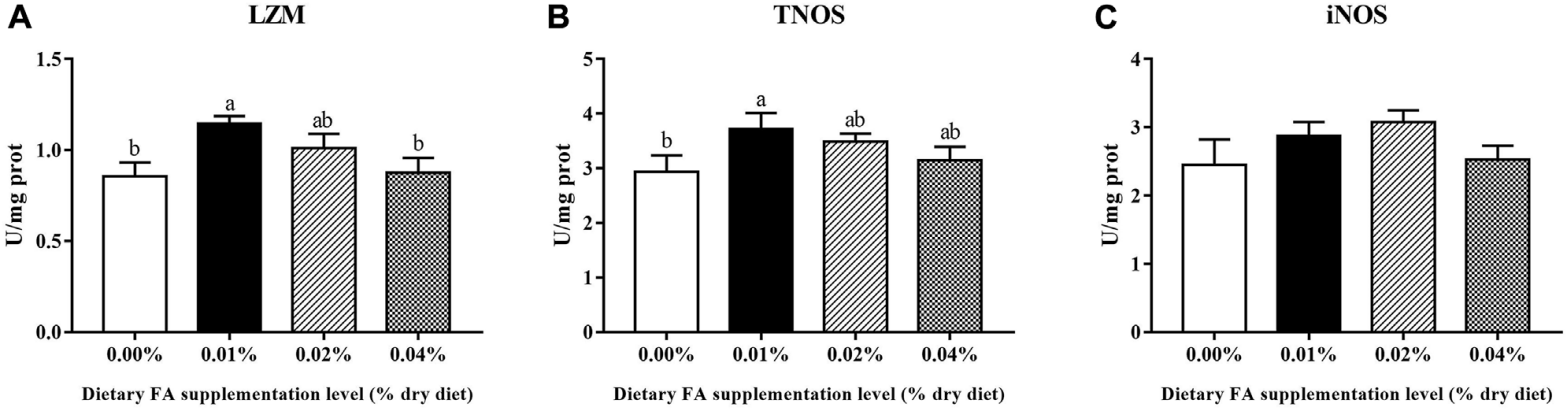
Effects of supplemental fulvic acid on LZM **(A)**, TNOS **(B)** and iNOS **(C)** activities in the visceral mass of large yellow croaker larvae (mean ± S.E.M, n = 3). LZM, lysozyme; TNOS, total nitric oxide synthase; iNOS, inducible nitric oxide synthase. Bars with different letters showed a significant difference (*p* < 0.05, Tukey’s test).

Supplementation of 0.01% FA significantly increased the enzyme activities of LZM and TNOS (*p* < 0.05) (Figure 4A, B). The supplemental FA increased iNOS activity, but the change was not statistically significant (*p* > 0.05) (Figure 4C).

### 3.6 Expression of inflammation cytokines genes

**FIGURE 5:**
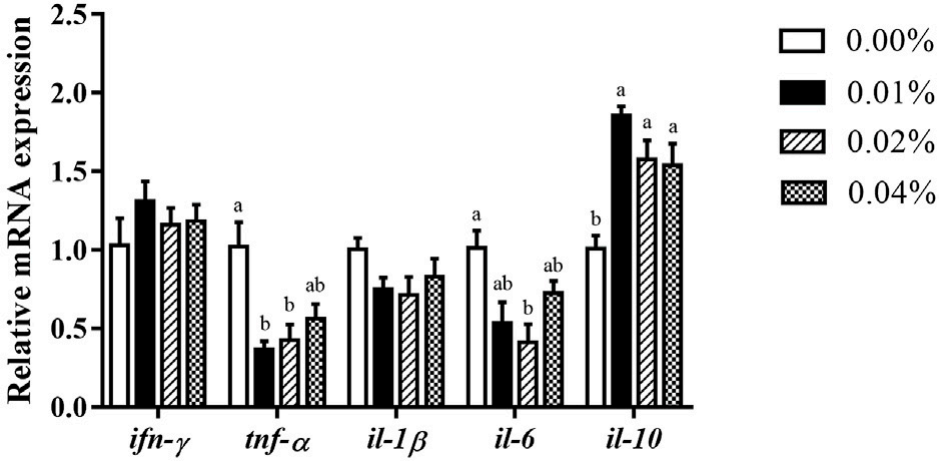
Effects of supplemental fulvic acid on genes related to inflammation mRNA expression in liver of large yellow croaker larvae (mean ± S.E.M, n = 3). *Ifn-γ*, interferon γ; *tnf-α*, tumor necrosis factor α; *il-1β*, interleukin-1β; *il-6*, interleukin-6; *il-10*, interleukin-10. Bars with different letters showed a significant difference (*p* < 0.05, Tukey’s test).

With an increase in FA supplementation, the mRNA expression of pro-inflammation cytokines *tnf-α*, and *il-6* decreased initially, then increased (Figure 5). When FA supplementation achieved 0.04%, no significant difference was found in the mRNA expression of *tnf-α* (*p* < 0.05). Supplementation of 0.02% FA significantly decreased the mRNA expression of *il-6* (*p* < 0.05)*.* Simultaneously, the supplementation of FA significantly increased the mRNA expression of *il-10* (*p* < 0.05) (Figure 5). There was no significant difference in the mRNA expression of *ifn-γ* and *il-1β* among all groups (*p* > 0.05) (Figure 5).

## 4 Discussion

FA is one of innocuous and eco-friendly organic substances, which has been proved to effectively reduce feed conversion rate and promote the growth of livestock and aquatic animals as a feed additive (Bai et al., 2013; Gao et al., 2018; Mao, 2019). In this study, the supplementation of FA could increase the survival and growth of larvae. The similar results have also been found in Nile tilapia (Jusadi et al., 2020) and Pacific white shrimp (Gao et al., 2018). These positive effects are related to the ability of FA to improve digestive capacity and immunity with its multiple active functional groups.

Intestinal development of marine fish larvae is important for improving digestive capacity (Pérez-Sirkin et al., 2020). In the present study, the intestinal VH and MT of larvae were significantly increased, indicating a more developed intestinal structure than that of the control group. Intestinal maturation is related to the proliferation and barrier function of epithelial cells (Liu et al., 2020). The mRNA expression of epithelial cell proliferation related gene (*pcna*) was significantly upregulated by supplementation of 0.01% FA. The permeability of tight junctions plays a crucial role in regulating the barrier function of the intestinal epithelium. This barrier allows for the absorption of nutrients and water while effectively blocking the entry of pathogens (Turner, 2006; Elsabagh et al., 2018; Li et al., 2021). In the present study, we found that the mRNA expression of intestinal *zo-1* and *zo-2* was significantly increased in the treatment group. Gildea et al. (2017) found that 20% lignite extract (the main raw material for FA extraction) was able to alleviate damage to the tight junction protein ZO-1 caused by glyphosate. Consequently, supplemental FA could improve intestinal maturation of larvae by ameliorating intestinal morphology, stimulating intestinal epithelial cell proliferation and maintaining intestinal barrier function.

Digestive enzyme activities are widely used as the marker to assess the digestive capacity of larvae (Yao et al., 2020; Yin et al., 2022). The relatively low activity of protease enzymes is thought to be one of the causes of the poor growth of larvae fed on a micro-diets (Dabrowski, 1984; Segner et al., 1989; Kolkovski, 2001). The present study showed that supplemental FA significantly increased the trypsin activities of IS and PS, which was consistent with the results in *P. clarkia* (Zhang, 2018), *P. dabryanus* (Gao et al., 2017) and *C. auratus* (Wu et al., 2016). The increased and stable activity of intestinal BBM enzymes such as aminopeptidase and AKP indicates that the gut may fully mature and achieve full function at this stage of development (Kolkovski et al., 2009; Ma et al., 2005; Lallès, 2020). The present study found that supplemental FA could significantly improve AKP and LAP activities in the intestinal BBM, which means that FA promote the intestine maturation of larvae (Liu et al., 2020; Xu et al., 2022). Thus, larvae fed diets with FA improved digestive enzyme activity and promoted intestinal development, facilitating adequate digestion and absorption of nutrients in the feed sufficiently.

The innate immune system plays a vital role in the immune defense of fish. Effective prophylactic measures to enhance the innate immune system prior to infection are of special value in larviculture (Magnadottir, 2010; Rojo-Cebreros et al., 2018). Oral immunostimulants can increase stress resistance and stimulate potential killing activity of macrophages to defend themselves against pathogens (Geng et al., 2011). Our research found that supplemental FA effectively improved activities of non-specific immune enzymes. LZM is involved in an extensive range of defense mechanisms and can act on the peptidoglycan layer of bacterial cell walls leading to bacterial lysis (Saurabh and Sahoo, 2008). Therefore, the increased activity of LZM indicates an improved protection against bacterial invasion. In vertebrates, Nitric oxide (NO) produced by immune cells is synthesized and released for over time by iNOS, which has unique flexibility in responding to disease (Huang et al., 2020; Pederzoli and Mola, 2016). In RAW246.7 cells, FA regulate iNOS production and stimulate NO production by activating the NF-κB pathway (Jayasooriya et al., 2016). In our study, the increased activity of iNOS indicated that FA had an immunostimulatory effect on larvae. Moreover, FA acts as an immunomodulator and has anti-inflammation effect (Sherry et al., 2013; Van Rensburg et al., 2001). Cytokines, as signaling molecules for cellular communication, are key participants in the innate immune response (Magnadottir, 2010). In the present study, FA stimulation led to upregulation of anti-inflammatory cytokine *il-10* and downregulation of pro-inflammatory cytokines *tnf-α* and *il-6*. Therefore, our research suggested that supplemental FA can enhance innate immunity of larvae.

In conclusion, the present study showed that the supplementation of FA can improve the survival rate and growth performance of large yellow croaker larvae by improving intestinal morphology, promoting intestinal barrier function, increasing digestive enzymes activities, and enhancing non-specific immunity. Based on the specific growth rate, the supplementation of 0.0135% FA was optimal for the growth performance of large yellow croaker larvae under the current experimental conditions.

## Data Availability

The original contributions presented in the study are included in the article/supplementary material, further inquiries can be directed to the corresponding author.
